# Effects of Intratesticular Lidocaine in Pet Rabbits Undergoing Orchiectomy

**DOI:** 10.3390/ani14040551

**Published:** 2024-02-07

**Authors:** Matteo Serpieri, Giuseppe Bonaffini, Chiara Ottino, Giuseppe Quaranta, Mitzy Mauthe von Degerfeld

**Affiliations:** Centro Animali Non Convenzionali, Dipartimento di Scienze Veterinarie, Università degli Studi di Torino, Largo Paolo Braccini 2, 10095 Grugliasco, Italy; matteo.serpieri@unito.it (M.S.); giuseppe.bonaffini@unito.it (G.B.); giuseppe.quaranta@unito.it (G.Q.); mitzy.mauthe@unito.it (M.M.v.D.)

**Keywords:** anesthesia, intratesticular, lidocaine, local anesthetic, orchiectomy, rabbit

## Abstract

**Simple Summary:**

Castration is a routine surgical procedure performed on rabbits to prevent reproduction. The use of balanced anesthesia for surgeries is crucial to reduce the doses of anesthetic drugs and their associated adverse effects. The addition of a local anesthetic can further enhance anesthetic performance. The aim of this study was to evaluate the effects of intratesticular lidocaine administration in rabbits undergoing orchiectomy, comparing it with a control group treated with saline solution. Rabbits administered lidocaine exhibited a reduced incidence of responses to surgical stimuli, and a diminished postoperative pain score was observed through evaluation using a composite pain scale, compared to the rabbits administered with saline. No clinically significant adverse effects related to lidocaine were detected. Consequently, the use of intratesticular lidocaine may be considered to improve the anesthetic performance of rabbits undergoing elective orchiectomy.

**Abstract:**

The use of local anesthetics for castration is both simple and cost-effective, and it may contribute to reducing the anesthetic requirements. Despite its common use in clinical practice, the literature regarding the effects of intratesticular lidocaine in rabbits is limited. In this study, nine rabbits per group were assigned to intratesticularly receive either 2% lidocaine (0.05 mL/kg into each testicle) or an equivalent volume of saline prior to elective orchiectomy. Anesthesia was induced by intranasal administration of ketamine, medetomidine, and butorphanol. During intraoperative assessment, no significant differences in vital parameters (heart rate, respiratory rate, and peripheral saturation of oxygen) were observed between the groups. However, rabbits receiving intratesticular saline displayed a higher incidence of responses to surgical stimuli. Postoperative pain was evaluated using the composite Centro Animali Non Convenzionali Rabbit Scale (CANCRS), revealing a significantly lower score at the initial post-surgery assessment in rabbits treated with intratesticular lidocaine. All subjects exhibited rapid resumption of food intake and fecal output. While all rabbits demonstrated satisfactory perioperative performances, the use of intratesticular lidocaine was associated with a diminished response to surgical stimuli. Consequently, this practice has the potential to reduce the requirement for additional anesthetics or analgesics, promoting faster recovery.

## 1. Introduction

Surgical neutering is a routine procedure that most pet rabbits undergo to prevent reproduction and the development of unwanted behaviors and hormone-induced diseases [1,2]. In veterinary medicine, orchiectomy is typically performed under general anesthesia, with an anesthetic level sufficient to prevent autonomic and motor responses related to the surgical stimulus [3,4]. To avoid adverse effects related to high doses of anesthetic drugs, it is preferred to combine multiple classes of drugs, reducing doses and achieving balanced anesthesia. Additionally, the use of local anesthetics during castration is generally simple and cost-effective, further reducing anesthetic requirements and minimizing response to surgical stimulus. Furthermore, local anesthesia is a common practice in veterinary medicine in the form of intratesticular or intrafunicular injections [3,4,5].

Among local anesthetics, lidocaine is the one of the most widely used in veterinary medicine for its fast onset time, its moderate duration of action, and its moderate toxicity [6]. Lidocaine is an amino-amide commercially available in injectable formulations and applicable topically. It contributes to multimodal analgesia by blockade of sodium channels in sensory nerve fibers, thereby suppressing the activity, amplitude, and conduction of electrical impulses [6,7].

Effective perioperative pain management is imperative in rabbits, even beyond the surgical period, as nociception may trigger sympathetic nervous system activation. This could exert adverse effects on tissue perfusion, immune response, wound healing, and gastrointestinal motility. Consequently, this can lead to delays in the resumption of food intake and fecal output after an anesthetic event [8,9,10]. Given their status as prey species, rabbits often do not overtly manifest pain in numerous instances. Untreated pain could compromise welfare and delay recovery, creating stress in both the pet and the owner; for this reason, the use of analgesic drugs in the perioperative period is mandatory in conditions of pain [10,11]. The utilization of composite scales tailored for pain assessment in rabbits, such as the Centro Animali Non Convenzionali Rabbit Scale (CANCRS), proves beneficial for evaluating pain through the analysis of facial expressions, physiological parameters, and behavioral cues. This tool can help the clinician in treating the possible presence of pain [12,13].

Given that the administration of intratesticular local anesthetics, including lidocaine, has the potential to mitigate pain and nociceptive responses linked to castration, this approach is commonly employed and advised in clinical settings for rabbit orchiectomy [14,15]. Nevertheless, there is a lack of published studies addressing the specific application of intratesticular lidocaine in rabbits.

Therefore, the objective of the current study was to assess the impact of intratesticular lidocaine, in comparison to saline treatment, in pet rabbits undergoing orchiectomy. This evaluation involved the analysis of vital parameters and response to the surgical stimulus during the intraoperative period. Additionally, the study employed the CANCRS composite scale to assess pain and further compared the times of resumption of food intake and fecal output in the postoperative period.

## 2. Materials and Methods

The study protocol was approved by the Ethical committee of the Department of Veterinary Science of the University of Turin, protocol n. 0004179/2022.

Power of 0.80, alpha error of 0.05, and 25% increase in mean heart rate (set at 160 ± 30 beats/min, based on the mean heart rate after anesthetic induction in rabbits using the same protocol as this study [16]; see Supplementary Materials) in non-treated animals compared to treated ones were considered for sample size calculation. Results indicated that the use of at least 18 rabbits (9 per group) would prevent a type II error (www.clincalc.com, accessed on 10 November 2022).

The study included 18 client-owned pet rabbits of various ages, weights, and breeds, undergoing elective orchiectomy, and signed written informed consent was obtained from the owners. Obese, cachectic, and ill subjects were excluded. Only rabbits displaying clearly visible testes within two scrotal sacs and exhibiting no clinically detectable anatomical alterations of the gonads prior to the surgical procedure were included.

### 2.1. Surgical and Anesthetic Procedures

The subjects were admitted to the hospital a day prior to the procedures to minimize the stress associated with transportation and to acclimate the rabbits to the environment. No food or water restrictions were imposed before the anesthesia. Baseline (T0) heart rate (HR) and respiratory rate (RR) were determined through thoracic auscultation and observation of chest movements, respectively.

For the anesthetic induction, a combination of 20 mg/kg ketamine (Lobotor^®^ 100 mg/mL, Acme S.r.l, Corte Tegge-Cavriago, Reggio-Emilia, Italy), 0.4 mg/kg medetomidine (Dormisan^®^ 1 mg/mL, ATI Azienda Terapeutica Veterinaria S.r.l., Milan, Italy), and 0.2 mg/kg butorphanol (Nargesic^®^ 10 mg/mL, Acme S.r.l, Corte Tegge-Cavriago, Reggio-Emilia, Italy) was intranasally administered using a Mucosal Atomization Device (MAD) (MAD Nasal™ Intranasal Mucosal Atomization Device MAD300, Teleflex Medical S.r.l., Varedo, Monza-Brianza, Italy), following the procedure described by Mauthe von Degerfeld et al. [16].

After the loss of the righting reflex (LRR), each rabbit was placed on a heating pad in dorsal recumbency and connected to a multiparameter monitoring system (Infinity Delta^®^, Dräger Italia SpA, Corsico, Italy) to monitor vital parameters via electrocardiography (ECG) and pulse oximetry. Rabbits received 1.5 L/min of 100% oxygen through a face mask (Anesthetic face mask, S, Jørgen Kruuse A/S, Langeskov, Denmark) using a non-rebreathing respiratory system (Bain coaxial breathing system, Intersurgical, Wokingham, UK).

The rabbits were randomly assigned to two groups (www.randomizer.org, accessed on 1 April 2023). In group L (lidocaine), 1 mg/kg lidocaine 2% (Lidocaina 2%, 20 mg/mL, Ecuphar Italia S.r.l., Milan, Italy), equivalent to 0.05 mL/kg, was intratesticularly (IT) injected into each testicle (total dose: 2 mg/kg), following inspection for blood aspiration with gentle negative pressure applied. In case of blood aspiration, the injection was discontinued and performed again at an immediately adjacent point, following the same procedure. For IT administration, each testicle was palpated and held in the scrotal sac by the operator using the left hand, while the injection was administered with the right hand. The filled syringe with a 25 G needle attached was inserted into the testicle perpendicular to its major axis, penetrating up to the midpoint of the gonad. In group S (saline), the same procedure was performed, injecting 0.05 mL/kg sterile saline (Sodio Cloruro 0.9%, S.A.L.F. S.p.A. Laboratorio Farmacologico, Cenate Sotto, Bergamo, Italy) into each testicle. The administered solutions were kept at room temperature (20–22 °C).

At least two minutes passed before proceeding with the surgery. During this time frame, the hair surrounding the testicle was clipped, and surgical scrub was performed using chlorhexidine (LH New Chlorhexidine 70, Lombarda H S.r.l., Abbiategrasso, Milan, Italy).

Orchiectomy was conducted using a scrotal approach by an open–closed technique [17]. The order in which the procedure was performed (beginning with the left or right testicle) was randomly assigned to each rabbit (www.randomizer.org). All surgical procedures were performed by the same surgeon (G.B.).

In the event of a 20% increase in HR, or sudden increase in RR, isoflurane was delivered (1%; IsoFlo, Zoetis Italia S.r.l., Rome, Italy) using a vaporizer (Vapamasta 6, Anmedic AB, Vallentuna, Sweden), and the surgical procedure was interrupted for 30 s.

At the end of the surgical procedure, 2 mg/kg atipamezole (Sedastop^®^ 5 mg/mL, Ecuphar Italia S.r.l., Milan, Italy) was intranasally administered using the MAD, and 1 mg/kg meloxicam (Meloxidyl^®^ 5 mg/mL, Ceva Salute Animale S.p.A., Milan, Italy) was subcutaneously (SQ) administered for postoperative pain management. After reoccurrence of palpebral and pedal withdrawal reflexes, each rabbit was placed in a cage prepared with soft blankets for recovery. An infrared heating lamp (InfraRed Industrial Heat Incandescent, Philips Lighting, Signify Italia S.p.A, Milan, Italy) was attached to each cage and maintained for 30 min. The rabbits were monitored until the resumption of the righting reflex (RRR, calculated from administration of atipamezole). Hay, rabbit pellet food, and fresh vegetables were provided after the righting reflex was regained.

All rabbits received 5 mg/kg enrofloxacin SQ (Baytril^®^ 25 mg/mL, Elanco Italia S.p.A., Sesto Fiorentino, Italy) once a day for five days.

### 2.2. Perioperative Evaluations

Five minutes after drug administration, a modified numerical sedation score (ranging from 0 to 12) for rabbits was compiled by evaluating posture, palpebral and pedal reflexes, resistance to physical restraint, and response to fur clipping (Table A1), as described by Raekallio et al. [18] and Santangelo et al. [19].

During the surgical procedures, HR, RR, SpO_2_, and responses to surgical stimulus (RSS; gross movements, or retraction of testicles) were recorded at the following 6 time points: during the incision of the tunica vaginalis of the first testicle (T1), during the detachment of the caudal attachment of the ligament of tail of the epididymis from the cremaster (T2), and during the ligation of the spermatic cord (T3). The parameters were evaluated in the same manner and at the same time points for the other testicle (T4, T5, T6).

The assessment of postoperative pain presence was based on the CANCRS composite scale [12]. Evaluations were conducted at specific time points: prior to the surgical procedure (S0), 6 h post-surgery (S1), and on the subsequent day at three time points (at 9:00 am (S2), at 1:00 pm (S3), and at 6:00 pm (S4)).

For each rabbit, the times to resumption of food intake and fecal output were recorded. Time was measured in hours from RRR, by hourly visual inspections of each rabbit and its cage. These inspections focused on identifying the presence of fecal pellets and determining if the rabbit was eating or had consumed the available food.

Consistent with the CANCRS scale assessments, the same designated operator (M.S.) conducted the evaluations, while Veterinary Medicine students were responsible for assessing the times of resumption of food intake and fecal output.

### 2.3. Statistical Analysis

Data management and statistical analysis were performed with Microsoft Excel (Microsoft 365, 2023; Microsoft Corp., Redmond, WA, USA) and R (version 4.2.2; R Foundation for Statistical Computing, Vienna, Austria). Continuous variables were not normally distributed (Shapiro–Wilk test with *p* < 0.05). Therefore, variables are reported as median and interquartile range, and non-parametric statistical tests were used.

Two-tailed Wilcoxon rank sum test and Fisher’s exact test were performed when applicable to evaluate homogeneity between groups L and S for the following variables: weight, age, duration of surgery, RRR, time from IT injection to surgery, total sedation score, and preoperative CANCRS score (S0).

Two-tailed Wilcoxon rank sum test and Fisher’s exact test were performed when applicable to compare time to resumption of food intake, time to resumption of fecal output, total CANCRS scores at each time point (S1 to S4), RRS, and HR, RR, and SpO_2_, at each intraoperative time point (T1–T6) between the groups.

Friedman’s test and subsequent Wilcoxon signed rank test with Bonferroni correction were performed to evaluate total CANCRS scores at each time point (S1 to S4), and HR, RR, and SpO_2_ at each intraoperative time point (T1–T6) within the groups.

Statistical significance was set at *p* < 0.05.

## 3. Results

No rabbit was excluded from this study.

The results regarding weight, age, total sedation score, duration of surgery, RRR, time from intratesticular administration to surgery, RSS, and times of resumption of food intake and fecal output in males are reported in Table 1.

RSS (i.e., slight movements of retraction of the testicles) occurred in group S in 3/9 rabbits at T1 and in 1/9 rabbits at T4 and at T5. No response occurred in group L. No gross movements occurred in any groups.

Events of a 20% increase in HR or sudden variations in RR, requiring isoflurane delivery, did not occur in both groups at any time point.

The results regarding differences in heart rate (HR, beats/min), respiratory rate (RR, breaths/min), and peripheral saturation (SpO_2_, %) between group L and S at each intraoperative time point are reported in Table 2.

The results regarding final CANCRS scores at each time point are reported in Table 3.

Statistically significant differences in final CANCRS scores over time were found within each group following Friedman’s test (L: *p* = 0.030; S: *p* = 0.038), but no differences within groups for each parameter were found following Wilcoxon signed rank test with Bonferroni correction (*p* = 0.462 for S2 vs. S5 in group S, and *p* = 1.000 for all other comparisons for both groups).

No differences were found in HR, RR, and SpO_2_ within group L (HR, *p* = 0.392; RR, *p* = 0.766; SpO_2_, *p* = 0.423). No differences were found for the same parameters within group S (HR, *p* = 0.175; RR, *p* = 0.990; SpO_2_, *p* = 0.997).

No blood aspiration occurred in any case before IT injection.

## 4. Discussion

The results provide promising insights into the potential benefits of incorporating intratesticular lidocaine in rabbit orchiectomy. The increased number of responses to surgical stimulation in group S suggests a localized antinociceptive effect exerted by intratesticular lidocaine in group L. Despite the widespread use of intratesticular lidocaine and its assessment across various species [3,4,5,20,21,22], a comprehensive understanding of its mode of action is not fully known. Following the intratesticular administration of radiolabeled lidocaine in two horses, substantial uptake into the spermatic cord was evident, indicative of a localized effect [20]. It is presumed that upon injection into the testicle, the local anesthetic flows through the testicle via lymph vessels, diffusing to block nerve fibers in the spermatic cord [23].

Although the potential for a systemic effect of lidocaine cannot be ruled out, it is improbable that the employed dosage would lead to plasma concentrations resulting in clinical consequences. This hypothesis is also supported by the results of previous studies. In horses subjected to intratesticular lidocaine, a notable portion of the drug has been shown to persist in the excised testes [20]. Also, a study involving adult cats revealed that a 2% lidocaine solution administered topically on the larynx at 0.1 mL, in addition to 0.1 mL/kg administered intratesticularly, resulted in serum concentrations well below toxic levels known for this species [24]. In fact, the limited systemic absorption also supports the safety of intratesticular administration, as local anesthetics can have toxic effects systemically, particularly on the central nervous and vascular systems, with the potential occurrence of seizures and severe arrhythmias. Such effects typically occur in the event of accidental intravenous injection, possible during blind administration within the testicle, given the presence of important vascular structures such as the pampiniform plexus [6,25]. An assessment of the safety of intratesticular lidocaine in rabbits would be of significant relevance, as a previous study reported seizures following intravenous lidocaine administration at doses of 5–6 mg/kg [26]. In the current study, the total dose of lidocaine was lower (2 mg/kg). The dose is equivalent to that recommended in a previous study for intravenous administration in a bolus, followed by a constant rate infusion at 100 μg/kg/min as additional analgesic treatment in rabbits undergoing ovariohysterectomy. No adverse effects were reported in the mentioned study after the bolus injection [27]. Furthermore, in this study, the aspiration procedure before intratesticular administration allows for the exclusion, in most cases, of accidental intravenous injection [25]. Nevertheless, since even mild negative pressure application could cause vein collapse, in the event of potential needle insertion into a vessel, partial intravenous injection cannot be entirely ruled out. Therefore, the veterinarian must be alert in detecting any potential onset of adverse effects. Although no pharmacokinetic studies have been conducted following intratesticular lidocaine injection in rabbits, it is probable that the drug predominantly manifests its effects at a local rather than systemic level, even in this species, without inducing systemic clinical consequences.

Beyond the absence of the onset of neurotoxic or cardiovascular adverse effects directly attributable to lidocaine, the lack of statistically significant differences in vital parameters between and within the groups suggests that intratesticular lidocaine can be administered without compromising the overall physiological stability of rabbits. This result is consistent with findings in other species, where intratesticular lidocaine use did not induce relevant cardiocirculatory effects [3,4,5,20,21,22]. In contrast, in male cats undergoing orchiectomy, intraoperative heart rate variability was lower in subjects treated with intratesticular lidocaine, presumably reducing hemodynamic variations induced by castration [28]. A limitation of the present study is the lack of measurement of arterial blood pressure, a valuable parameter in the intraoperative assessment of nociception. This parameter was not measured in this study due to the unavailability of the module for pressure measurement in the monitor in use at that time at the facility where the procedures were conducted.

It was decided to wait at least two minutes before proceeding with the surgery after intratesticular administration, based on the onset time of lidocaine (2–5 min) [29]. However, the patient preparation required a slightly extended period, and the surgery was performed several minutes after the injection (median: 10 min in group L, and 8 min in group S), ensuring adherence to the onset time of the local anesthetic. The choice to administer lidocaine in both testicles before surgery was due to the rapidity of elective orchiectomy, which would have allowed for intervention within the duration of action of lidocaine (about 2 h) [29,30]. The duration of surgery (median: 13 min in group L, and 9 min in group S) was shorter than this time, and the beginning of the surgery on the first testicle was still beyond the onset of the action of lidocaine. Therefore, there should not have been any differences during the removal of the first and second testicles.

Postoperative evaluation using the CANCRS composite scale revealed a significant difference between the groups at the first assessment following the surgical procedure, with a lower final score in group L. Despite this difference, the observed median values were low in both groups, well below the cutoff value of 7, previously identified as indicative of abdominal pain and the need for analgesic treatment [31]. This finding suggests that the anesthetic protocol and the use of meloxicam for postoperative analgesia resulted in adequate pain management. Intratesticular lidocaine may have exerted an additional residual local analgesic effect, contributing to a slightly lower postoperative CANCRS score in group L. Indeed, the excision of the testicles through gonadectomy leads to the simultaneous removal of most of the injected local anesthetic. However, it is possible that during the intraoperative period, lidocaine may spread in the surrounding tissues through its flow via lymph vessels, as hypothesized to explain the effects following intratesticular administration [6]. This phenomenon could contribute to establishing a residual effect of the local anesthetic in the peritesticular soft tissues involved in the surgical procedure. Moreover, in addition to local anesthetic properties, lidocaine appears to also exert anti-inflammatory effects, although the mechanisms and duration of action are not yet fully understood. However, it is assumed that the drug impacts various inflammatory processes as phagocytosis, migration, exocytosis, and cellular metabolism [32]. This could have contributed to reducing the pain mediated by inflammation in the immediate postoperative period, due to the spread of the drug in the surrounding tissues. In addition to what has already been mentioned, the use of local anesthetics in the context of preventive analgesia can also be considered. The local anesthetic inhibits intraoperative nociception and the consequent stimulation of the central nervous system. This, in the majority of studies, contributes to reducing the need for analgesic drugs in treated subjects compared to a placebo group [33,34,35]. Consequently, intratesticular lidocaine may have played a role in pain management during the early postoperative phase for all these reasons in this study.

Effective perioperative pain and nociception management are further supported by the fact that all animals showed a rapid resumption of food intake and fecal output, crucial parameters in the postoperative assessment of rabbits [8,9,10], and no significant differences were observed in these parameters. Although, as mentioned above, it is unlikely that lidocaine absorption occurred at concentrations causing clinical effects, this drug seems to have a positive impact on situations of anorexia and gastrointestinal stasis in rabbits. Administered intravenously, lidocaine has been shown to improve prognosis by stimulating the resumption of proper intestinal motility in rabbits and horses, preventing motility alterations through its anti-inflammatory, anti-endotoxic, or pain-relieving actions [27,36,37]. Thus, a potential partial absorption of lidocaine could even contribute to the postoperative recovery of normal gastrointestinal activity, reducing hospitalization times.

Intratesticular injection is often correlated or compared with intrafunicular administration, especially in livestock animals. The rationale is the blockade of sensory activity directly at the level of the spermatic cord near the nervous and vascular structures, proximal or at the site of vessels and duct ligation, and incision for gonad excision [21,23,38]. Although, as reported in a recent study, injection at the level of the funiculus seems to provide a postoperative analgesic effect by reducing IL-6 and TNF-α [38], this technique is more easily performed in species with a pendulous scrotum, such as horses and ruminants, where it is routinely executed and investigated [23,38]. The rabbit testicle, on the other hand, is located in a scrotal sac that only partially contains it, allowing for easy palpation of the gonad itself but not of the spermatic cord, which is often surrounded by a variable amount of fat, making its identification more complex through palpation [39,40]. The choice to administer lidocaine intratesticularly, therefore, was based on avoiding iatrogenic damage to structures involved in surgery. The preference was to administer the drug into the gonad that would be removed. This was accomplished through a simple and rapid manual procedure, repeatable by personnel with limited experience with the specific anatomy of the rabbit.

Based on the obtained results, the use of intratesticular lidocaine for rabbit orchiectomy leads to a reduced response to surgical stimuli compared to a placebo group using an equivalent anesthetic protocol, potentially allowing for a reduction in the doses of administered drugs. The responses observed in group S were slight retractions of the testicles during surgical manipulation, never accompanied by significant alterations in the clinical markers of nociception considered—in this case, heart rate (HR) and respiratory rate (RR)—or gross movements. This suggests that, nonetheless, the anesthesiological protocol used would have been adequate for the surgical procedure, ensuring anesthetic stability. In our study, the choice of using a Mucosal Atomization Device to administer the anesthetic drug combination was made, as it was found to produce a lighter anesthetic plane compared to intramuscular administration of the same combination, resulting in faster recovery [16]. A relatively light anesthetic plan would have allowed for a more effective detection of a response to the surgical stimulus in the subjects. Although the use of a combination of anesthetics with minimal or no analgesic effects would have been optimal for detecting nociceptive responses, the choice to use a mixture of ketamine, medetomidine, and butorphanol, drugs with analgesic effects, has ethical considerations, respecting animal welfare. The choice to intervene with the use of isoflurane only, although it does not have analgesic properties, was based on previous studies evaluating the isoflurane-sparing properties of lidocaine [5,7]. However, an intervention was never required, probably due to the analgesic effect of the used drugs. Moreover, although fentanyl is usually employed as a rescue analgesic, it is often not readily available in private practice and could cause respiratory depression [3]. For these reasons, the choice was to utilize a simple anesthetic protocol that could also be used easily in clinical practice. The use of this protocol, combined with intratesticular lidocaine, appeared suitable for effective perioperative pain management in orchiectomy, ensuring rapid recovery and subsequent discharge of client-owned rabbits. Thus, the use of lidocaine could allow for a further reduction in the doses of anesthetic drugs, thereby minimizing adverse effects in the context of a balanced multimodal anesthesia approach.

A limitation of the current study was the lack of postoperative patient evaluation through cameras. While direct assessment of rabbits has proven reliable using the CANCRS [12], it is acknowledged that employing cameras for remote patient assessment minimizes stress associated with the presence of an observer. This factor could potentially influence the scores obtained using the utilized scale [11]. As mentioned above, the lack of blood pressure measurement is a limitation of the study and could be a useful parameter to employ in future research. Additionally, it would be beneficial to assess lidocaine absorption and plasma concentration after intratesticular administration, considering the occurrence of adverse effects related to neurotoxicity in rabbits in a previous study [26]. Lastly, a comparison of the ease of execution and potential adverse effects resulting from intrafunicular administration compared to intratesticular administration in rabbits could further enhance the anesthesiological practices employed during orchiectomy in this species.

## 5. Conclusions

Intratesticular injection of 1 mg/kg lidocaine 2% prior to orchiectomy in rabbits resulted in a reduced response to surgical stimuli compared to a placebo treatment. No other intraoperative parameter, including HR, RR, and SpO_2_, was affected by the treatment. Both lidocaine- and saline-treated rabbits exhibited effective perioperative pain management, with a rapid resumption in food intake and fecal output. The administration of intratesticular lidocaine represents a simple and cost-effective procedure. When combined with an appropriate anesthetic protocol and postoperative analgesia, it may decrease the need for additional treatment, promoting faster recovery and reducing hospitalization times.

## Figures and Tables

**Table 1:** Differences in weight, age, total sedation score, duration of surgery, RRR, IT injection to surgery, response to surgical stimulus, and times of resumption of food intake and fecal output in rabbits treated either with preoperative IT lidocaine (group L) or IT saline (group S). Results are reported as median (interquartile range).

Parameter	Group L	Group S	*p* Value
Weight (kg)	1.70 (1.47–1.96)	1.63 (1.46–1.70)	0.691
Age (months)	10 (6–13)	12 (8–22)	0.564
Total sedation score (0–12)	11 (10–12)	11 (10–11)	0.964
Duration of surgery (min)	13 (9–14)	9 (9–10)	0.185
RRR (min)	43 (28–45)	33 (28–56)	1.000
Intratesticular injection to surgery (min)	10 (8–15)	8 (8–9)	0.528
Response to surgical stimulus (events, %)	0/9 (0%)	5/9 (55.5%)	0.029 *
Resumption of food intake (h)	7 (5–7)	8 (3–11)	0.860
Resumption of fecal output (h)	6 (5–10)	3 (2–9)	0.289

* Statistically significant difference.

**Table 2:** Differences in heart rate (HR, beats/min), respiratory rate (RR, breaths/min), and peripheral saturation (SpO_2_, %) between group L and S. Results are reported as median and range.

	Time Points					
Variable	T1	T2	T3	T4	T5	T6
HR (beats/min)						
Group L	180 (166–200)	180 (160–200)	175 (160–190)	170 (162–190)	170 (160–190)	166 (160–180)
Group S	172 (160–187)	174 (160–193)	170 (156–191)	180 (166–196)	178 (166–196)	170 (166–196)
*p* value	0.480	0.626	0.923	0.566	0.596	0.630
RR (breaths/min)						
Group L	32 (24–68)	32 (24–68)	40 (27–71)	28 (24–68)	28 (24–68)	38 (24–71)
Group S	66 (42–66)	66 (42–66)	60 (42–66)	48 (48–60)	48 (48–60)	48 (44–60)
*p* value	0.595	0.565	0.810	0.505	0.505	0.847
SpO_2_ (%)						
Group L	100 (100–100)	100 (100–100)	100 (100–100)	100 (100–100)	100 (100–100)	100 (100–100)
Group S	100 (99–100)	100 (97–100)	100 (97–100)	100 (98–100)	100 (98–100)	100 (98–100)
*p* value	0.456	0.214	0.265	0.076	0.076	0.098

**Table 3:** Comparison of CANCRS scores between groups in rabbits treated either with preoperative IT lidocaine (group L) or IT saline (group S). Scores are reported as median (interquartile range, IQR).

CANCRS Score (Min–Max)	Group L	Group S	*p* Value
S0 (0–24)	3 (3–3)	3 (3–4)	0.450
S1 (0–24)	2 (2–3)	4 (3–4)	0.031 *
S2 (0–24)	3 (3–5)	3 (2–5)	0.830
S3 (0–24)	2 (2–3)	3 (2–4)	0.304
S4 (0–24)	2 (2–2)	2 (1–2)	1.000

* Statistically significant difference.

## Data Availability

The raw data supporting the conclusions of this article will be made available by the authors on request.

## Supplementary Materials

The following supporting information can be downloaded at: https://www.mdpi.com/article/10.3390/ani14040551/s1.

## Appendix A

**Table A1:** Modified numerical sedation score for rabbits [18,19].

Parameter	Behavior of the Rabbit	Score
Spontaneous posture	Normal	0
	Lying sternally, head up	1
	Lying sternally or laterally, responding to stimuli	2
	Lying, not responding to stimuli	3
	Complete muscle relaxation	4
Palpebral reflex	Normal	0
	Decreased	1
	Absent	2
Response to fur clipping	Normal response	0
	Reduced	1
	Absent	2
Resistance to physical restraint	Normal resistance	0
	Moderate resistance	1
	No resistance	2
Pedal reflex	Normal	0
	Decreased	1
	No reaction	2
Total	Insufficient	0–3
	Moderate	4–7
	Deep	8–12
